# Practitioners' Bookshelf

**Published:** 1891-10-24

**Authors:** 


### PRACTITIONERS' BOOKSHELF.

### ATLAS OF CLINICAL MEDICINE.*

The first two parts of Volume I of Dr. Bramwell's "Atlas of Clinical Medicine" promise well for the great work he has taken in hand. The Atlas is to be issued in four parts yearly and each part is to consist of 32 folio pages, and the whole work will include at least three yearly volumes. The chief feature of the publication is the illustrations; those of Part I and II are most artistic and reproduce in colour the characteristics of the diseases represented most faithfully. Thus Part I contains plates of Myxœdema, Sporadic cretinism and lymphadenoma, and Part II, Addison's disease, Hodgkin's disease, Molluscum fibrosum, and Xerodema pigmentosum. But the text is also a valuable part of the work; it is not simply a description of the plates but contains clinical histories of cases figured, and also articles on medicine, clinical and systematic, with woodcut illustrations. Dr. Bramwell's name and reputation is sufficient guarantee that the succeeding parts will be in keeping with the earlier instalments which leave nothing to be desired.

*Atlas of Clinical Medicine. Vol. I., Part I and II. By Byrom Bramwell, M.D., F.R.S.E. Edinburgh: T. and A. Constable. 1891.

